# Nopal Cactus (*Opuntia Ficus-Indica*) as a Holographic Material

**DOI:** 10.3390/ma5112383

**Published:** 2012-11-20

**Authors:** Arturo Olivares-Pérez, Santa Toxqui-López, Ana L. Padilla-Velasco

**Affiliations:** 1 Optical Department, National Institute of Astrophysics Optics and Electronics (INAOE), Luis Enrique Erro No.1, Tonantzintla, Puebla 72840, Mexico; 2 Engineering Faculty, Autonomous University of Puebla, Puebla 72000, Mexico; E-Mails: stoxqui72@hotmail.com (S.T.-L.) and, anapadi21@yahoo.com.mx (A.L.P.-V.)

**Keywords:** holographic material, diffraction gratings, holography, pheophytins, biopolymer, nanosciences, chlorophyll

## Abstract

The nopal cactus (*Opuntia ficus-indica*) releases a substance through its mucilage, which comes from the degradation of pectic substances and chlorophyll. Combined in a polyvinyl alcohol matrix, this substance can be used as a recording medium. The resulting extract material has excellent photosensitizer properties, is easy to handle, has a low cost, and low toxicity. This material has the property of self-developing, and it can be used in holographic applications. The polyvinyl alcohol and extract from the nopal cactus was deposited by a gravity technique on a glass substrate, which dried to form a photosensitive emulsion. We show experimental results on a holographic grating using this material, written by a He-Cd laser (442 nm). We obtained diffraction gratings by transmission with a diffraction efficiency of approximately 32.3% to first order.

## 1. Introduction

Photopolymers can record information with self-developing, without a chemical process for obtaining a holographic image. These materials haves achieved high recording capacity, light sensitivity, durability, resolution, and longer life for stored information [1,2,3].

The development of photopolymer materials has become one of the most dynamic developing branches of holography [1,2,3,4,5]. Holographic recording with organic polymer materials has been studied intensively in order to achieve high efficiency, low cost, and high storage density [6,7,8].

Polyvinyl alcohol (PVA), although not directly involved in the photochemical reaction, is an important factor due its high sensitivity to humidity and environmental changes. These properties are crucial for the polymeric matrix and significantly affect the physical conditions of the recording medium such as rigidity, environmental stability, and changes upon holographic exposure [9,10]. In addition, PVA has been used to make holographic films, which has been doped with different dyes for oxidant stimulating birefringence [11]. In this manuscript, along with other preliminary results [12], we show how PVA as a polymeric matrix sensitized with the mucilage from the nopal cactus and chlorophyll degradation can be used in holographic recording material. 

The manuscript is organized as follows: in Section 2, “Materials,” we describe the nopal cactus (*Opuntia ficus-indica*), including its origins, mucilage, chlorophyll, and its use as a photobiopolymer. In Section 3, “Methods,” we present a technique for photosensitive dye preparation. We describe a process for obtaining a photopolymer layer with PVA and show some chemical characteristics of the photosensitive dye by UV-visible spectroscopy and infrared vibrational infrared spectroscopy. Furthermore, we show the experimental results for the diffraction efficiency parameter of holographic gratings, the optimum exposure energy, and the spatial frequency of diffraction gratings to vary the registration angle. The work ends with the conclusions in Section 4.

## 2. Materials

### 2.1. The Nopal Cactus (Opuntia Ficus-Indica)—Origins

The nopal cactus (*Opuntia ficus-indica*) may have a hybrid origin through human manipulation. It is an historical plant studied in several documents indicating the possibility that hybridization was achieved through human pollination, or was accidentally mixed in an indirect way. This may have occurred indirectly, including allopathic sympatric ancestors cultivated from central Mexico [13]. One possibility is that hybridization occurred in the ancestral lands of Mesoamerica, where crops of *O. ficus-indica* were cultivated directly by farmers. There is evidence of selective farming of crops of these plants by pre-Columbian peoples [14]. Mesoamerican plant reproduction technology may have included pollination, as in other ancient cultures [13,15].

### 2.2. Mucilage 

The mucilage of the nopal comes from the degradation of pectic substances, which are biopolymers composed of polysaccharides such as pectins. Such a composition is useful as a potential feedstock for the production of edible plastic films [16,17]. The efficiency of an edible film made from cactus mucilage has been proven for coatings on strawberries (*Fragaria ananassa*). An increase in lifespan was observed without affecting the color and flavor of food [18], using the extracting pectin with gelling a capacity and a nongelling mucilage fraction from *O. ficus-indica* cladodes [18,19]. The sugar composition indicates that all the polysaccharides obtained contain anionic moieties, and galacturonic acid residues [20]. The mucilage isolated from *O. ficus-indica*, contains arabinose galactose, galacturonic acid, rhamnose and xylose [21].

Studies of the degradation of mucilage have been made by various techniques through the analysis of different processes. The partial hydrolysis of the mucilage of *O. ficus-indica* has been shown by methylation analysis and periodate oxidation [22]. Analysis of the polymeric mucilage after incubation with the fungal culture has also been studied [23].

### 2.3. Chlorophyll 

Chlorophyll is the photoreceptor pigment responsible for the first stage in the transformation of the energy of sunlight into chemical energy. Chlorophyll is found in specific organelles, chloroplasts, associated with lipids and lipoproteins [24]. There are two main types of chlorophyll, chlorophyll “a” and chlorophyll “b”. Chlorophyll “b” has a formyl group (-CHO), instead of the methyl group (-CH_3_), found in chlorophyll “a”, in one of the carbons in the porphyrin ring. In higher plants the most abundant chlorophyll is the “a” type [24,25].

Chlorophyll is the photosynthetic pigment in all plants, and a fluorescent molecule, its presence can be determined by fluorometry [5,26]. Chlorophylls “a”, “b”, “c1”, “c2”, “d” and “f” and their pheophytins are quite different in both their excitation and emission wavelengths. Each pair has its own acid ratio. A careful study of the acid ratios of various mixtures of pure chlorophylls a and c shows this effect [26,27,28]. 

Chlorophyll is what gives the green appearance to the cactus *O. ficus-indica*, and with the production of mucilage, both provide an important role in the decomposition of cactus. Due to its high nutrient content, the decomposition process is accelerated and produces marked changes in the coloration of the leaf of the cactus [29,30]. 

The green color of immature leaves and fruits is due to chlorophyll “a” (bluish green) and chlorophyll “b” (yellow-green) in a 3:1 ratio. By removal of magnesium, the chlorophylls are transformed into pheophytins “a” and “b” which are olive drab. The substitution of the Mg++ ion, can be performed by the following metal ions by Fe++, Sn++, Zn++ and Cu++, which results in the formation of brown-gray products. Heating at elevated temperatures and shorter times maintains the color better than when heating for longer times and lower temperatures [27,30,31]. 

**Figure 1:**
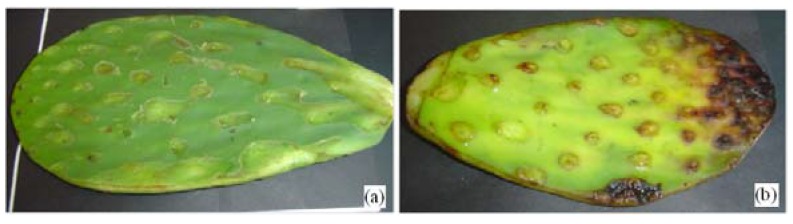
(**a**) Fresh nopal cactus (*Opuntia ficus-indica*); (**b**) Observe the color change from green to dark brown in the cactus.

Figure 1 shows a change of color in the cactus cladode. Figure 1a shows the yellowish-green color of a fresh cactus cladode due to chlorophyll ratio 3:1. Figure 1b shows the cactus cladode after several days, where color change begins from yellow-green to dark brown due to the decomposition of the mucilage and chlorophyll. Mg ions contained in the core of the chlorophyll begin to be replaced by other metal ions.

### 2.4. Chemical Composition (Opuntia Ficus-Indica)

The Nopal, which is highly nutritional, has in its chemical composition a large variety of molecular specie, which varies in number of molecules per species, caused by the aging of the samples [32,33,34,35]. Among the most important organic species detected in samples are: carbohydrates, protein, fiber, fat, and ash [32,33,34,35]. Its most important mineral species include: phosphorus, manganese, iron, zinc, magnesium, calcium, potassium, and sodium [32,36,37]. In smaller amounts, it also contains: lithium, vanadium, cobalt, arsenic, selenium, cadmium, thallium [32,37]. There is evidence that some elements of the chemical composition depend on different factors, such as pH, water availability, soil texture and mineral composition where the cactus grows. Organic species contained in the sample contain hydroxyls from phenolic compounds, which are released in the decomposition process, favoring oxidation of some of the mineral elements. The enzymatic browning is due to the oxidation of phenolic compounds by the enzyme polyphenoloxidase (PPO) [38,39].

### 2.5. Photobiopolymer

The substitution of Mg++ by Fe++, Sn++, Zn++ and Cu++ from pheophytins reactions, results in a potentially photosensitive biopolymer, with the ability to record holographic or photographic information when used as the carrier matrix of photosensitive metal ions with the PVA [40]. Some work has been reported using this matrix mixed with salts of ferric chloride (FeCl_3_). The basic recording mechanism in such samples is attributed to the photo-crosslinking process of PVA when mixed with metallic salts. During the photo-crosslinking process there are two different Fe pathways: and active Fe^3+^ and an inactive Fe^2+^ [41,42,43]. Salts of copper (II) chloride dihydrate CuCl_2_ (2H_2_O) show photosensitivity and the ability to conduct electricity. Due to the nature of copper, there are generally photo-redox processes tightly surrounding the components, promoting strong crystallization. The fact that copper chloride dihydrate allows for easy assimilation into a matrix of PVA is used to build our conductive and photosensitive film [44,45], copper doped with methylene blue [46], holograms with porphyrins using Zn doped with nematic liquid crystals, and hydrogels PVA-HG [47,48].

## 3. Methods

### 3.1. Cactus (Opuntia Ficus-Indica) Dye Preparation

A clear brown solution is obtained from the fermentation of the nopal cactus; this solution was used as a photosensitizer, as obtained from enzymatic reactions that occur when the cactus is stored inside a box (no light) at a temperature of 10 °C.

The solution is stored and, after a few days, it is possible to observe enzymatic browning and mucilage drainage due to chlorophyll degradation. This phenomenon becomes evident already in a 24-hour period. The entire process of chlorophyll transformations from nopal occur during storage to obtain the clear brown solution, with a minimally processed cactus, over the course of 12 to 18 days at 10 °C [39,49].

Therefore, we can determine that cactus’s life (under conditions of environmental or cooling) due to three deterioration reactions that occur simultaneously:

(a) The changing of the cactus’s natural color to dark green and then brown is due to enzymatic reactions in which the enzyme responsible is polyphenol-oxidase [35,38,39];
(b) The drainage of mucus (slime);
(c) The rapid growth of microorganisms (bacteria, fungi and yeasts).

The polyphenol-oxidase (PPO) found in “nopal” is responsible for enzymatic browning reactions occurring during storage. The polyphenol-oxidase, also known as tyrosine, or cresolas phenoloxidases, catalyzes the hydroxylation of monophenols to ortofinoles, which are subsequently oxidized to orthoquinones. The resulting polymerized pigments exhibit a brown color [35,38,39].

(1) Nopal cactus (*Opuntia ficus-indica*) is cut into small slices (1 cm × 1 cm about), while removing the prickly thorns, and is then placed in a container (Nopal weighing 250 g can produce an approximate quantity of 12 mL fermented extract).
(2) The cactus is then stored in a plastic container that is sealed and cooled to 10 °C for 10 to 18 days, to obtain an optimal fermentation and get the clear brown solution. On the other hand, with a minimally processed cactus, after one or two days at room temperature (30 °C), it will already demonstrates signs of fermentation.
(3) Deterioration of the cactus in the vessel was observed when the mucus became brown colored. This solution is then filtered by a vacuum filtration method.
(4) After filtering, the extract has a shelf life of five to ten days (at room temperature). In that period, it is appropriate to prepare photosensitive plates. (After these days at room temperature, using the extract to a photosensitive film, the surface of the film shows its deterioration as cracks in the surface).
(5) The solution may be stored under refrigeration (5 °C and 10 °C) for 30 or 60 days.
(6) One of the conditions of this solution for use as a photosensitizer is pH. Due to the variation of pH as a function of storage time and temperature, we worked with a pH of less than seven (Conductronic model PH-10). Normally, fermentation substances tend to decompose and produce alcohols, which lead to an acidity in the substance with a pH less than seven.

This known characteristic of pheophytins and this phenomenon is known as phosphating. Since phosphating reaction rates are generally higher than other chlorophyll degradation pathways, this is considered an important mechanism of chlorophyll destruction during the storage of nopal. This chemical reaction induces a structural transformation of chlorophyll in the nopal that replaces the magnesium group by other metallic ions. A clear brown solution is obtained from the fermentation of nopal cactus (Table 1). This solution is filtered before it can be used to sensitize the polymeric PVA matrix. 

**Table 1:** Summary of some observed properties of the extract from fermented nopal cactus.

Properties	States
Physical state	Liquid
Appearance	Clear brown/dark Brown
Solubility	Water, alcohol
Stability condition	Color change with time
pH	acidic (pH < 7)

### 3.2. Photopolymer Layer Preparations

Our use of PVA as a matrix to contain the fermented cactus extract is suitable due to its high hygroscopic capacity. PVA is also easy to mix with the fermented mucilage. Polyvinyl alcohol (Baker^®^) is partially hydrolyzed (87.0%–89.0%), with a viscosity of 4% aqueous, and the alcohol has a powdered form.

The technique for making photosensitive layers with PVA and nopal cactus extract can be summarized in five processes:

(1) The entire mixture is obtained at room conditions in the laboratory, with the average temperature of 20 °C; and a relative humidity of about 40%.
(2) Polyvinyl alcohol (Baker^®^) was prepared in a 12% solution with distilled water at a temperature of 85 °C.
(3) PVA (Baker^®^) is mixed with nopal cactus extract in a proportion of 2.5 mL and 0.8 mL (32%). This proportion was found to be the optimum value during the experiments.
(4) The PVA layers doped with nopal cactus extract were prepared by pouring a small amount of the solution onto a glass slide by gravity technique. After 24 hours of storage in complete darkness under normal laboratory conditions (room temperature 20 °C, relative humidity 40%) the samples are dried.
(5) The dry film having 40 µm thickness, with a extract (nopal) concentration at (32% V/V), by pouring 0.5 mL of solution on a substrate area of 9 cm^2^. This was measured with a digital micrometer (Mitutoyo Corporation^®^ Model IP65). The thickness of the photosensitive film may be modified by varying the amount of solution poured onto a certain area on the substrate surface.

### 3.3. Absorption UV-Visible Spectrum 

**Figure 2:**
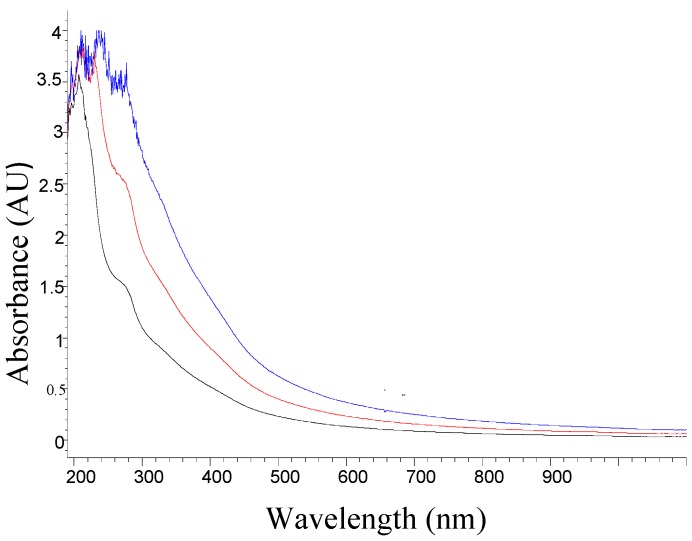
Absorbance UV-Visible spectrum of the photosensitizer mixed with PVA at three different concentrations, without dilution (blue), 50% diluted (red), and 75% diluted (black).

Films were prepared for holographic recording using the PVA polymer matrix and the resulting solution from the nopal cactus as a photosensitizer. We determined the wavelength corresponding to the maximum absorption for the mixture of PVA plus the photosensitizer solution using a spectrophotometer (Hp model 8453). Figure 2 shows the absorption UV-Visible spectrum for the mixed saturated solution of PVA (12%) with photosensitizer (extract at 100%).

The proportions shown below refer to the amount of PVA solution that was prepared (12%), and which always remains constant. More the amount of cactus extract at 100%, this would correspond to the ratio (1:1). The other proportions represent the addition of distilled water to ratio (1:1) to obtain 50% (V/V) and 75% (V/V). 

Photosensitizer concentrations were varied, without dilution (proportion 1:1, blue line), 50% diluted (red line), and 75% diluted (black line). The PVA polymer matrix was prepared. Figure 2 shows that the absorbance increases more rapidly at shorter wavelengths than in the longest wavelengths, when the concentration increases. As also shown in Figure 2, the wavelength response is more suitable for working in the near UV region, and between 400 nm and 700 nm which corresponds to the visible region.

**Figure 3:**
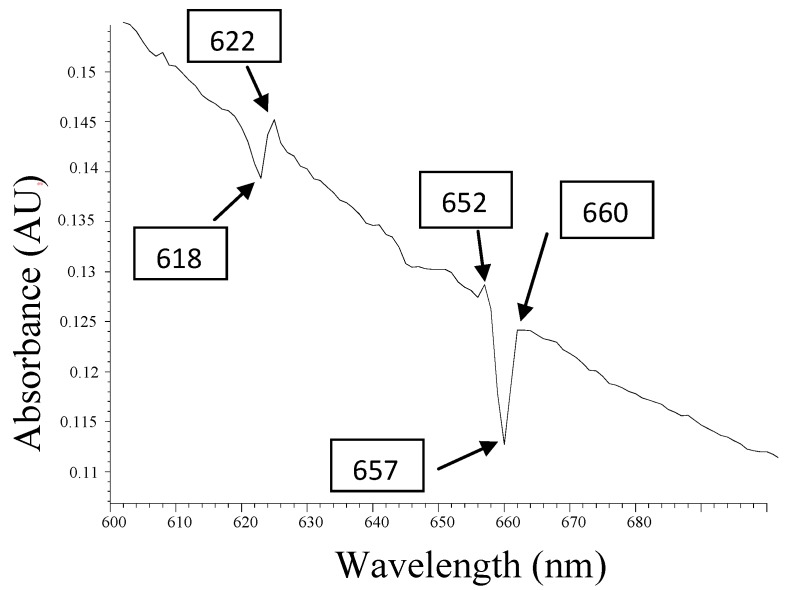
Absorbance analysis of UV-visible spectrum, which shows two small valley areas that illustrate the absorption changes centered at 618 nm and 657 nm, thus corresponding to the phaeophytins, Q band.

The absorbance of the photosensitive film in Figure 2 shows larger values of absorbance in the UV region. A point of note is that by varying the concentrations between the extract of nopal and PVA, the absorbance values in the visible spectrum begin to increase, thereby giving the photosensitive material the ability to record holograms in other wavelengths. For example, the proportion 75% (dark line) can work optimally in the 220 nm line. However, 100% (blue line) can work optimally from 220 nm to 340 nm. We work with a He-Cd laser at 442 nm, showing an increase in absorbance by a factor of three times (see Figure 3), due to changes in concentration that correspond to four times, with respect to the initial ratio (1:1). This concentration (75%) is the most optimal of these graphs (Figure 2) for photosensitive films, and recording holographic diffraction gratings.

All samples were worked with the same thickness 40 μm, which is determined by the amount of substance poured on a given surface area of the substrate used.

### 3.4. UV-Visible Details Analysis

Derivatives of the constituents from the nopal cactus mucilage and chlorophyll degradation are usually determined through analysis of the UV-visible region.

The graph in Figure 3 shows an atypical behavior of chlorophylls, with respect to other works reported, due to the high content of metallic minerals in the cactus extract [32,37]. However, small changes in absorbance in the region Q, in the valleys at 618 nm and 657 nm, show an activity of this phenomenon, which, in this case, is derived from the decomposition of chlorophyll. The extract contained minerals, as described in Section 2.4; some minerals can form pheophytins from the iron and zinc. What catches one’s attention is the position of the edges of these valleys: 622 nm, 652, 660 are very close to the values reported of phaeophytins typical of iron [50,51]. The 622 nm region corresponds to Fe-pheophytin and the 652 nm region corresponds to pheophytins. The 660 nm region corresponds to chlorophyll in the Q region (absorption band red spectral regions) from the UV-visible spectrum [52,53,54]. These small changes of absorption peaks in the Q region between 600 nm and 700 nm indicate the presence of Fe^2+^ ions.

The importance of this result shown in Figure 3 is the detection of Fe^2+^ ions, by analyzing absorption UV-Visible spectrum. Small changes in amplitude as shown in the absorbance spectrum in the region Q, are primarily due to the amount of biomass reactions involved in the extract of the fermented material, which form the pheophytins, supplied by Fe^2+^ ions. In other words, reactions which generate a substitution of chlorophyll core of the Mg++, by a metal Fe++ ion, form a photosensitive substance. It is also expected that many Fe++ ions failed to form pheophytins, and other reactions were derived by resonant given the Fe^2+ ^ ions. In fermented extract biomass, there are a number of free hydroxyl ions, molecules from water, carbohydrates, sugars, alcohols, metal ions where some can make an oxidation transition of Fe++ to Fe+++ [55,56,57,58,59]. With all of these combinations of; pheophytins, due to their amplitude values expressed in Figure 3, it is understood that the conversion of pheophytins is not efficient. That is, in the process of photosensitization of the material, there are more mechanisms that stimulate this phenomenon involved. 

It is known that the nopal has the mineral composition of phosphorus, manganese, iron, zinc, magnesium, calcium, potassium, sodium [32,37]. A peculiarity of iron ion Fe++ (ferrous), is its ability to blend; it regularly combines with ions from bromide, chloride, oxide, carbonate, hydroxide, nitrate, phosphate, sulfate or dichromate [55,59]. Also generated as a result of the fermentation, are alcohols, acids, and sugars, which stimulate the production of hydroxides (OH) that promote the oxidation of Fe++ ion that then becomes Fe+++ (ferric) [55,56,57,58,59]. With respect to Table 2, the values are very small because of the small amount of Fe in the fermented biomass extract. Also, the minerals can interact among themselves, forming ionic groups [55,59]. It is understood that in the extract there is a complex group of minerals, which interact with the organic compounds, leading to greater photosensitivity.

The ratio is appropriate, given that its photosensitivity is very good, and because we can obtain excellent diffraction gratings with high diffraction efficiency.

**Table 2:** Pheophytins in the visible zone Q band.

Peaks(nm) Q band	Abs(AU)	Localization of the Pheophytins
622.0 nm	0.142	Fe-pheophytin
652.0 nm	0.125	Pheophytins
660.0 nm	0.121	Chlorophyll

In regards to other commercial materials, the cactus extract is competitive in the diffraction efficiency parameter. However, the stability and lifetime of the film still has limitations. Slavich commercial films with fine grain emulsions: VRP-M (green) and PFG-01 (red) show a diffraction efficiency greater than 45% using aqueous developers with chemicals to make them highly efficient. [60]. Fujifilm F-HL-10 is a panchromatic photosensitive emulsion with a diffraction efficiency of 45%, using a developing process [61]. On the other hand, the most advanced commercial holographic film on the market is Litiholo, based on a polymeric film RRT20, which has a diffraction efficiency of 99% and has the property of self-developing [62].

### 3.5. IR Analysis 

**Figure 4:**
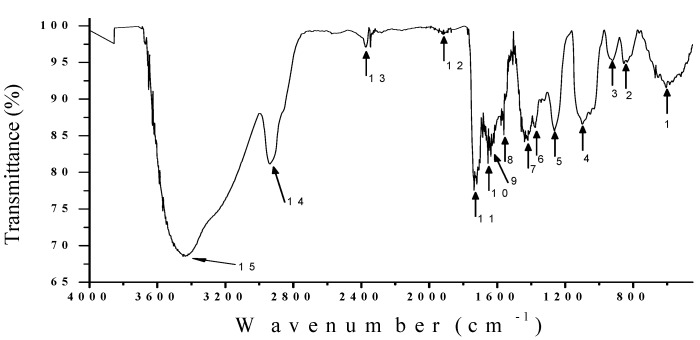
The vibrational behavior of the molecular structures that form the fermented extract nopal with the PVA through an IR analysis.

An IR spectroscopic study is essential for determining the vibrational states of molecular structures that form in the compounds or emulsions [63]. Figure 4 shows the infrared spectrum of the extract from the nopal mixture with PVA in liquid form. With a proportion of 2.5 mL and 0.8 mL. The dry film is similar to that used for recording holographic gratings having the same 40 µm thickness and solution concentration (32%). 

Figure 4 shows the infrared spectrum of the fermented nopal extract mixed with PVA, obtained with a Digilab-Scimitar® infrared spectrophotometer. Where is observed transmittance spectrum weighted (%) of organic compounds derived from the process of decomposition of chlorophyll and mucilage nopal cactus (*Opuntia ficus-indica*). All samples were manipulated in quartz containers, in order to obtain a vibrational spectrum, undisturbed by the vibrational links of the container. The infrared spectrum was obtained from a sample, with proportion of 2.5 mL and 0.8 mL (32%) using a liquid sample with the content of fermented extract. In Figure 4, we can see that the main organic compounds resonate at certain localized frequencies. Table 3 shows the most important elements of this vibrational spectrum corresponding to the organic compounds. The infrared spectrum presents a profile similar to previous work reported with water-soluble hemicellulosic fractions [64] and biomass derivatives from pine pellets [65].

Table 3 shows the IR spectra of a dry film of organic compounds derived from the process of decomposition of chlorophyll and the mucilage of nopal cactus. The hydroxyl group and ether signals (C-O-C) in sugar units are strongly absorbed at 3448, 2939, 1911, and 1099 cm^−1^.The bands at 2374, 1618, and 1560 cm^−1^ correspond to compounds derived from the degradation of chlorophyll by fermentation. In this case, it is common to observe some structures containing nitrogen such as amines and some amino acid residues.

**Table 3:** Organic elements produced by the fermentation of chlorophyll and mucilage, which were detected by their specific vibrational effects.

#	Vibrational bands (cm^−1^)	Probable Links
15	3448	N-H Amine, O-H Alcohol
14	2939	O-H Carboxylic Acid, N-H Amine, C-H Alkane
13	2374	N-H Amine
12	1911	C=C=C Alkene, \C=C/ Benzene ring, C=O Anhydride
11	1735	C=O Carboxylic Acid, C=O Ester, C=O Carbonyl, \C=C/ Benzene ring
10	1654	C=C Alkene, C-C Alkane acyclic
9	1637	C=C Alkene, N-H Amine, C-C Alkane monosubstituted
8	1618	N-H Amine, C=O Amino Acid zwitterions
7	1560	C=O Amino Acid, N-H Amine, \C=C/ Benzene ring
6	1419	C-H Alkane, N-O Nitro aliphatic
5	1263	C-O Carboxylic Acid, C=O Ester, C-H Alkane methyl
4	1099	C-O Alcohol, C-F Alkaly Halide, C-N Amine
3	921	=C-H Alkene, Benzene ring meta-disubstituted
2	854	=C-H Alkene, Benzene ring para-disubstituted
1	605	C-Cl Alkyl Halide, C-Br Alkyl Halide

Bands between 1263 and 1099 cm^−1^ are typical of arabinoxylans, due to the presence of the arabinosyl side-chains in the spectrum of holocellulose. The bands at 1654, 1419, and 1263 cm^−1^ in hemicellulose spectrum (spectrum a) originate from wagging vibrations of the carboxylate anion, methyl C-H, and carbonyl absorbance in pectic substances. These signatures confirm the hemicellulosic with pectic polysaccharide content, which corresponds to the results obtained from sugar and alcohol analysis.

Absorption at 1637 cm^−1^ in the spectrum of holocellulose usually has a strong affinity for water. In the solid state these macromolecules may have disordered structures that are easily hydrated. The small band at 1735 cm^−1^ in holocellulose is due to the acetyl, uronic, and ferulic ester groups of the polysaccharides. Bands between 1419 and 1099 cm^−1^ represent C-H and C-O stretching and CH or OH bending in polysaccharides. The bands from 921 to 854 cm^−1^ are holocellulose which is due to the C-1 group frequency or ring frequency characteristic of glycosidic linkages between the sugar units. The band at 605 cm^−1^ is derived from a process of degradation of chlorophyll as some halides, alkali and bromides.

The fermented extract combined with the PVA in laboratory conditions, thermally stable (20 °C) (relaxation time after 24 hours of storage, to perform measurements, and these measurements can be repeatable), which is represented in the IR spectrum of Figure 4. Depending on the environmental conditions of humidity and temperature occurs, an aging process of the photosensitive film (oxidation). This is due to the hydrophilic character of PVA.

### 3.6. Recording Process

**Figure 5:**
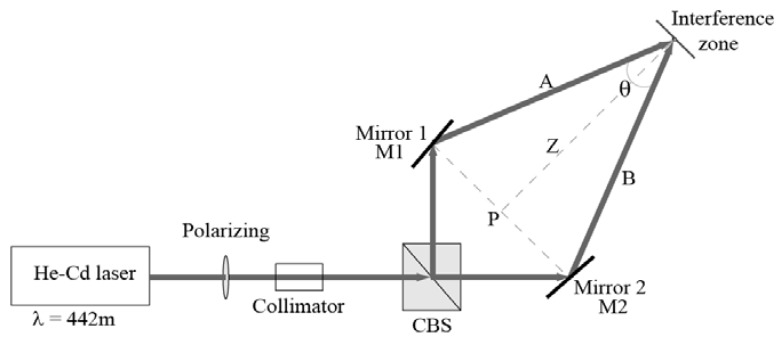
Experimental setup of symmetrical arms for the recording of holographic gratings. The recording beam wavelength was λ = 442nm [44].

Holographic diffraction gratings are recorded by placing the PVA and nopal cactus extract layer in an experimental setup. The optical setup for the holographic register is shown in Figure 5. We used a He-Cd laser at 442 nm (Ominichrome^®^ Series 56) for holographic recording with an output power up to 6 mW. The beam incident on the holographic material is linearly polarized. We fixed polarization using a linear polarizer on the laser output (polarization S). Both arms A and B are linearly polarized and have geometric symmetry (Where they form an isosceles triangle ABP). The main beam splits into two arms using a beam splitter prism. The angle formed between both arms is θ. The incident power is split equally between both arms. The two beams impinge at a point (interference zone) where an interference pattern forms. The power at this point is around 4.80 × 10^−3^ W/cm^2^. Holographic diffraction gratings are recorded, under normal laboratory conditions (room temperature 20 °C, relative humidity 40%). 

**Figure 6:**
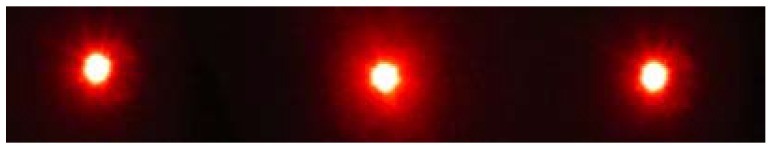
Optical reconstruction of the diffraction pattern from a holographic grating built from Figure 5.

**Figure 7:**
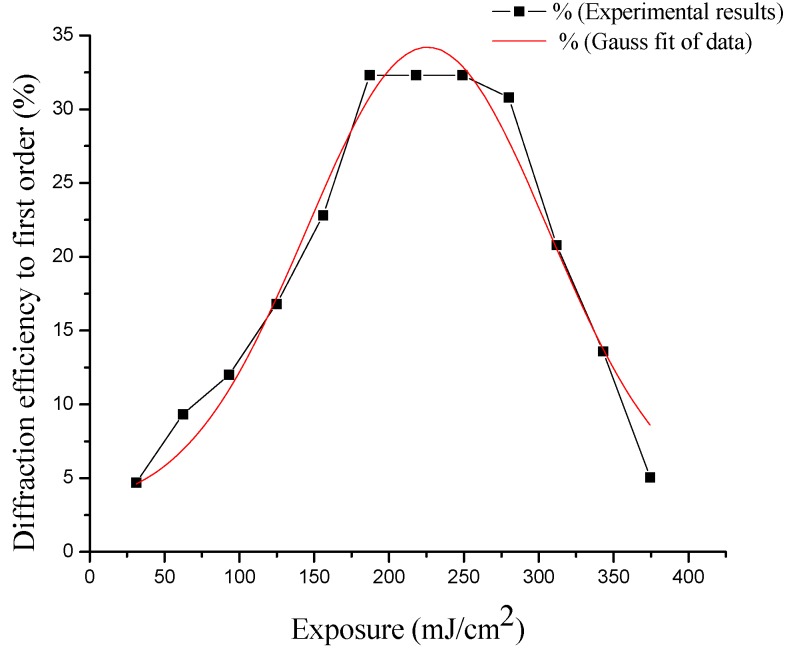
The experimental diffraction efficiency, with respect to recording energy from holographic gratings measured at the Bragg angle.

**Figure 8:**
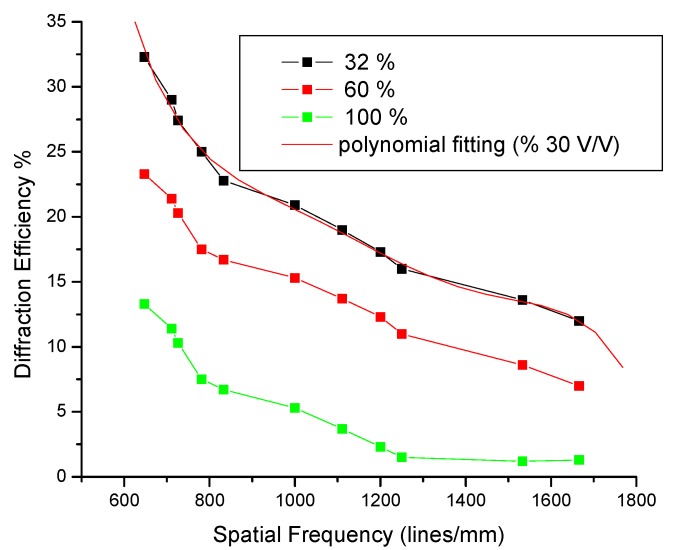
Behavior of a photosensitive polymer based on the nopal cactus extract, as a function of the spatial frequency of the holographic gratings recorded at three different concentrations.

This symmetry allows us to vary the distance Z between the interference zone and point P in order to obtain the diffraction patterns in Figure 6 and diffraction efficiency in Figure 7. Measurements were taken at several positions in order to have various angles leading to multiple frequencies for holographic gratings that are required to obtain the module transfer function (MTF) parameter shown in Figure 8, which is composed of 11 measurements. The size of the interference zone can be changed if we place a collimator before the prism system, working within an interference area of approximately 5 mm in diameter. Holographic gratings were recorded (646 l/mm, with a recording angle θ = 16.4 °) at different exposure times in Figure 7, which were reconstructed by a He-Ne laser (632. 8 nm).

### 3.7. Experimental Results

In this section are still doing studies and characterization of thickness and frequency of the diffraction grating, that soon we will send. But need the fermented extract reaches its maturity, to build diffraction gratings with different thicknesses and concentrations.

Figure 6 shows the optical reconstruction using a He-Ne laser at λ = 632 nm of the diffraction pattern of a sinusoidal grating obtained by holographic techniques. The orders 1+ and 1− have the same intensity of about 30%, slightly more than that zero order. However, diffraction efficiency is higher (~32.3%) at Bragg angles of the order 1+ or 1−.

The optical reconstruction was carried out directly without an expander laser beam. The laser beam is incident normally to the surface of the holographic grating. The intensity of the diffracted beams is then measured to first order. This value is divided by the incident laser beam intensity. In order to obtain the diffraction efficiency to first order, this quotient is multiplied by 100. To obtain and determine which diffraction regime, if it is Bragg or Ramman-Nath, the gratings is rotated from right to left to obtain the maximum efficiency of a diffracted order can be 1+ or 1−, at this angle is where it meets the regime type.

Experimental observations to determine the diffraction efficiency of PVA with nopal extract, for a dry film, using Kogelnik´s coupled wave theory Equation (1) [66].

(1) $$\eta =\sin{\left[\frac{\pi d\Delta n}{\lambda \cos(\beta )}\right]}^{2}$$

where η is the diffraction efficiency of gratings recorded in PVA with nopal extract; λ is wavelength of reading; d the thickness of sample; β is readout angle inside photomaterial; and Δn is the refractive index changes. 

The angle β is important, in photosensitive materials with substantial thickness. Because determines a preferred angle for maximum diffraction efficiency. In our case in Figure 6, the grating is reconstructed at normal incidence, thus the angle β has a value of zero. Then by rotating the grating from left to right, is located the preferred value of the angle β, and we obtain the Bragg angle. If only one of the diffracted orders significantly increases its intensity. Or determine whether it corresponds to Raman-Nath regime, if more orders of diffraction appear, or if the diffracted intensity is distributed among the other orders.

The material has a hybrid behavior, *i.e.*, has an amplitude modulation component (darkens the area irradiated with 442 nm, and the film shows a dark brown, depending on the nopal extract concentration), and phase modulation component (the material developed self, and by the thickness of the film). The diffraction gratings constructed holographically, were reconstructed using a readout beam with a zero angle (normal incidence) Figure 6. The intensities of the two beams are overlapped to form the interference zone were the same, to obtain a profile "sinusoidal". An important point is that we reconstruct the holographic gratings with He-Ne laser, because this wavelength has an absorbance less than 1%. Following Kogelnik's theory [66] and Klein-Cook criterion, which is based on sample thickness and the grating period, to determine if the system Raman-Nath or Bragg diffraction [67]. We observe that meets both regimes. 

The diffraction grating shown in Figure 5 has a thickness of 40 μm, and with spatial frequency of 646 lines/mm. we reconstruct the holographic grating at normal incidence where the orders are +1 and −1, with high diffraction efficiency, which does not correspond to a profile “sine”. Normally in these profiles zero order is much larger than the +1 and −1 diffracted orders, which would comply with the Raman-Nath regime [67]. In our case we note that the central order competes in energy with respect to the +1 and −1 diffracted orders, which fulfills the Bragg regime [66,67]. 

However, it is not necessary to know the thickness in order to determine which regime is operative [68]. 

### 3.8. Energy and MTF 

During the experiments a grating structure was recorded at various exposure times and from these results the diffraction efficiency average was calculated.

In Figure 7 the diffraction efficiency is plotted as a function of energy. The diffraction efficiency from each holographic grating array is characterized by taking the average of the two first orders of diffraction.

The diffraction efficiency from Figure 7 corresponds to gratings recorded into a layer prepared with 2.5 mL and 0.8 mL. (PVA/nopal extract) solution, which was found to be the optimum proportion (32%).

Furthermore, it is possible to observe the variation of the diffraction efficiency as a function of energy. As seen in Figure 7, the maximum diffraction efficiency of holographic gratings obtained with this material was 32.3% with exposure energy of the order of 223 mJ/cm^2^. These results were measured at the Bragg angle. The diffraction efficiency was measured with a He-Ne laser at 632 nm, externally by transmission. With this wavelength, the transmission loss effects are negligible, because the amplitude of absorbance of the material with the concentration and thickness is approximately 0.135 (AU). 

This distribution of the diffraction efficiency with respect to energy is typical of photosensitive Fe++ ion-based materials, where a distribution slightly skewed to the left is observe. The form of this distribution is retained, and can be obtained in several ways. Plotting, diffraction efficiency *vs.* exposure energy, or diffraction efficiency *vs.* exposure time [42,43]. We adjust this distribution with a Gaussian function for splines techniques, with the sole purpose of better visualizing the distribution and behavior of the experimental points obtained in measurements.

This material has the ability to record the information, and amplify the latent image without using a wet developed process; the material has the property of self-developing. Figure 7 shows the dynamic evolution of the recorded material as a function of exposure energy, given the optimal value of diffraction efficiency at 225 mJ/cm^2^. The exposure times depend on the registry laser output power, so it is more generally plotted with the exposure energy used.

Figure 8 was obtained by making various measurements through several positions in order to achieve various angles that in turn lead to multiple frequencies for holographic gratings, necessary for obtaining the MTF parameter. Up to 11 measurements were made for these graphs [69,70].

Figure 8 corresponds to the diffraction efficiency of the holographic gratings constructed in the arrangement shown in Figure 5 with 11 diffraction gratings for each concentration measurement. The concentration ratios of the solutions obtained from the nopal extract were: 2.5 mL PVA + 0.8 mL extract (32%), 2.5 mL PVA + 1.5 mL extract (60%), and 2.5 mL PVA + 2.5 mL extract (100%), while maintaining constant PVA solution. The points obtained from experimental diffraction gratings, resulting from a concentration of 32%, were adjusted by the technique of splines with the only purpose of better visualizing the distribution of the points in Figure 8. 

Table 4 shows the proportions of the substances that were used to record holographic gratings, observing a change in thickness due to the densities of the mixed materials. The concentrations of solution (PVA + nopal extract) determines the optical transparency of the film. When the film has more extract of nopal, the film will become darker (dark brown), reducing the diffraction efficiency of the order of 14.0% or less. 

**Table 4:** Concentration ratios.

PVA (mL ± 0.05)	Cactus Extract (mL ± 0.05)	% V/V	Solution on 9 (cm^2^)	Thickness Physical (μm ± 0.5)
2.5	2.5	100%	0.5 mL	45
2.5	1.5	60%	0.5 mL	43
2.5	0.8	32%	0.5 mL	40

As seen in Figure 8, the most suitable concentration was 32%, due to the maximum value of diffraction efficiency. In all curves, there is a reduction in the diffraction efficiency of the holographic gratings, as a function of the spatial frequency of the diffraction gratings. Holograms recorded with this material can have a lifespan of several months, depending on environmental conditions. The value of the diffraction efficiency of holograms decreases with time due to the natural aging process of the material. However, if the material is protected, its lifespan is much greater. The holograms can be protected with a glass surface, acetate, wood varnish, nail varnish, or epoxies. Such material has no ability to erase and rewrite information, such as the commonly used photorefractive crystals. In this case, the holograms are self-developing. 

## 4. Conclusions

The extract of fermented nopal cactus mixed with PVA has been shown to be an excellent material for building transmission holographic gratings. Temperature has an important role in the decomposition rates of chlorophyll and mucilage, through pheophytins reactions. The substitution of Mg atoms by the ion Fe ++ is highly likely in most nuclei of the chlorophyll, which results in the dark brown color from the chlorophyll fermented mucilage. The photosensitivity as shown in Figure 7 has a typical profile. Several studies have written holograms with Fe (III), which makes a transition to Fe (II) for recording an image. Another important indication of the presence of Fe ++ is the analysis of the UV-visible spectrum of the material. Here, two transition regions exist, as shown in Figure 3, demonstrating the presence of Fe-pheophytin, together with a mixture of combinations of many other mineral ions responsible for the photosensitivity of the material. The infrared spectrum shows the vibrational response of the molecules involved in the fermentation of chlorophyll and mucilage. The products indicate that a certain percentage of chlorophyll contained in this material has been altered by natural processes of fermentation through sugars and alcohols. The fermented extract is represented in the IR spectrum of Figure 4. Then, the PVA film with the fermented extract is dried. However, the extract is strongly affected by the environmental conditions of humidity and temperature, which determine the speed of the aging process of the photosensitive film (oxidation).

All these attributes make this self-developing material promising for recording a holographic image. Figure 7 shows the dynamic evolution of the recorded material as a function of exposure energy. Photosensitive material based on this extract behaves well, under normal laboratory conditions (room temperature 20 °C, relative humidity 40%). This material is easy to handle and the holographic elements constructed from this material have a diffraction efficiency of approximately 32.3% to first order at the Bragg angle. This photosensitive material presents a reduction of diffraction efficiency as a function of spatial frequency, as shown in Figure 8. The concentration ratio of the extract fermented nopal mucilage combined with PVA is a major factor for obtaining good photosensitive films. We found that a 2.5 mL PVA + 0.8 mL extract was optimum. Holographic elements built with this biopolymer should be feasible for future research work, in particular for finding ways to further increase diffraction efficiency and spatial resolution of the holographic gratings. 
